# Advances in Antisense Oligonucleotide Development for Target Identification, Validation, and as Novel Therapeutics

**DOI:** 10.4137/grsb.s418

**Published:** 2008-09-22

**Authors:** Moizza Mansoor, Alirio J. Melendez

**Affiliations:** Department of Physiology, Yong Loo Lin School of Medicine, National University of Singapore, Singapore

## Abstract

Antisense oligonucleotides (As-ODNs) are single stranded, synthetically prepared strands of deoxynucleotide sequences, usually 18–21 nucleotides in length, complementary to the mRNA sequence of the target gene. As-ODNs are able to selectively bind cognate mRNA sequences by sequence-specific hybridization. This results in cleavage or disablement of the mRNA and, thus, inhibits the expression of the target gene. The specificity of the As approach is based on the probability that, in the human genome, any sequence longer than a minimal number of nucleotides (nt), 13 for RNA and 17 for DNA, normally occurs only once. The potential applications of As-ODNs are numerous because mRNA is ubiquitous and is more accessible to manipulation than DNA. With the publication of the human genome sequence, it has become theoretically possible to inhibit mRNA of almost any gene by As-ODNs, in order to get a better understanding of gene function, investigate its role in disease pathology and to study novel therapeutic targets for the diseases caused by dysregulated gene expression. The conceptual simplicity, the availability of gene sequence information from the human genome, the inexpensive availability of synthetic oligonucleotides and the possibility of rational drug design makes As-ODNs powerful tools for target identification, validation and therapeutic intervention. In this review we discuss the latest developments in antisense oligonucleotide design, delivery, pharmacokinetics and potential side effects, as well as its uses in target identification and validation, and finally focus on the current developments of antisense oligonucleotides in therapeutic intervention in various diseases.

## Introduction

Antisense oligonucleotides (As-ODNs) are single stranded, synthetically prepared strands of deoxynucleotide sequences, usually 18–21 nucleotides in length, complementary to the mRNA sequence of the target gene. As-ODNs are able to selectively bind cognate mRNA sequences by sequence-specific hybridization. This results in cleavage or disablement of the mRNA and, thus, inhibits the expression of the target gene. The specificity of the As approach is based on the probability that, in the human genome, any sequence longer than a minimal number of nucleotides (nt), 13 for RNA and 17 for DNA, normally occurs only once [1].

The modification of gene expression, using a synthetic single stranded DNA, resulting in inhibition of mRNA translation was demonstrated for the first time by Paterson et al. in 1977 in a cell-free system [2]. Almost a year later, Zamecnik and Stephenson showed that in chicken fibroblast tissue culture containing Rous Sarcoma virus, addition of a synthetic13 mer ODN complementary to the 3′ terminal sequence of the Rous sarcoma virus, could inhibit viral replication and transformation of fibroblasts into sarcoma cells [3].

These seminal papers, and the thousands that followed, have caused enormous progress towards the development of a new class of drugs, As-ODNs.

**Figure 1:**
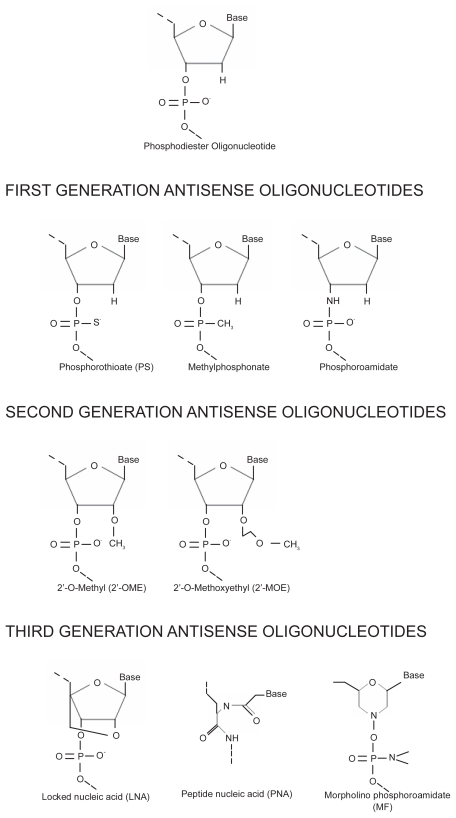
Structure of first, second and third generation antisense oligonucleotides.

The potential applications of As-ODNs are numerous because mRNA is ubiquitous and is more accessible to manipulation than DNA. With the publication of the human genome sequence, it has become theoretically possible to inhibit mRNA of almost any gene by As-ODNs, in order to get a better understanding of gene function, investigate its role in disease pathology and to study novel therapeutic targets for the diseases caused by dysregulated gene expression. The conceptual simplicity, the availability of gene sequence information from the human genome, the inexpensive availability of synthetic oligonucleotides and the possibility of rational drug design makes As-ODNs powerful tools for target identification and therapeutic intervention [4]. However, initial studies of As-ODNs, which utilized deoxyribonucleotides, were disappointing, as they were limited by a number of factors such as the poor solubility of ODNs, weak permeation across biological membranes and rapid degradation by endo and exo nucleases [5]. For As-ODNs to be therapeutically effective, it was necessary to improve their stability and efficacy while retaining their specificity. For this purpose several chemical modifications have been made to various components of As-ODNs, resulting in improvements in their stability, potency and bioavailability. On the basis of these modifications, As-ODNs can now be broadly classified into 3 generations: As-ODNs with altered phosphate backbone, those with modified sugars and those containing unnatural bases [6] (Fig. 1).

## Advances in the Design of Antisense Oligonucleotides

### First-generation antisense oligonucleotides

The first generation ODNs are synthesized by replacing one of the non-bridging oxygen atoms in the phosphate group with either a sulfur group (phosphorothioates), methyl group (methyl phosphonates) or amines (phosphoramidates). The first generation ODNs have more resistance to nucleases and longer plasma half life as compared with phosphodiester oligonucleotides. Moreover, they are easy to synthesize, carry negative charges that ease their cell delivery, are capable of activating RNAse H and have suitable pharmacokinetics [7]. At present, the phosphorothioate is the most widely used As-ODN modification and phosphorothioate oligonucleotides (PS-ODNs) have shown promising results both in vivo and in vitro. The only FDA approved As-ODN drug, Vitravene, and most of the other drugs in clinical trials are PS-ODNs.

However, as discussed to be later, first generation As-ODNs produce various undesirable, non-specific in vivo side effects, such as immune stimulation and complement activation, which are mainly caused by their interactions with proteins.

### Second-generation antisense oligonucleotides

To improve the binding affinity and hybridization stability with target mRNA and to increase the nuclease resistance, second generation As-ODNs, with alkyl modifications at the 2′ position of the ribose, were developed. The most commonly used second generation As-ODNs are 2′-O-Methyl (2′-OME) and 2′-O-Methoxyethyl (2′-MOE) ODNs. However, the useful effects of these modifications are dampened by the fact that they render 2′-OME and 2′-MOE incapable of activating RNAse H, an endonuclease whose recruitment is considered to be important for the activity of As-ODNs [8]. To induce RNAse H activation, chimeric As-ODNs were developed, by surrounding a central gap region consisting of a phosphorothioate deoxyribose core, with nuclease resistant arms such as 2′-OME or 2′-MOE. The resulting “gapmer” allows RNAse H to sit in the central gap to activate target specific mRNA degradation, while the arms, by virtue of alkyl modifications, prevent the degradation of As-ODNs by nucleases [9].

Second generation As-ODNS are reported to have a higher affinity for mRNA, better tissue uptake, increased resistance to nucleases, longer in vivo half life and lesser toxicity, as compared to first generation As-ODNs [10]. GEM 231 and GEM 92 (Hybridon) are examples of second generation As-ODNs currently being tested in the clinical trials.

### Third-generation antisense oligonucleotides

Third generation As-ODNs were developed by chemically modifying the furanose ring of the ODNs, along with modifications of phosphate linkages or of riboses, as well as of nucleotides. The modifications were made to improve the nuclease stability, target affinity and pharmacokinetic profiles of the ODNs. Locked nucleic acid (LNA), Peptide nucleic acid (PNA) and Morpholino phosphoroamidates (MF) are the three most commonly used third generation As-ODNs.

Third generation As-ODNs have higher stability in biological fluids as they are essentially resistant to degradation by nucleases and peptidases. They also exhibit a strong hybridization affinity with the mRNA. In addition, PNAs, due to their ability to recognize double stranded DNA, are capable of modulating gene expression or inducing mutation by strand invasion of chromosomal duplex DNA. However, third generation As-ODNs do not activate RNAse H and most likely produce their biological effects by causing steric hindrance of ribosomal machinery, resulting in translational arrest. Moreover, being uncharged molecules, they do not bind to serum proteins that normally bind poly anions. On the one hand, this reduces the likelihood of non-specific interactions, but on the other hand, hastens their clearance from the body. Also, their electrostatically neutral backbones make their solubility and uptake difficult [6]. To overcome these problems, in vitro, their delivery can be improved by employing the delivery systems mentioned later in this chapter.

Substantial data has demonstrated the effectiveness of third generation As-ODNs in various in vitro, ex vivo and in vivo models. PNAs, MFs and LNAs As-ODNs have shown promising results in various studies [11–13]. In addition, the efficacy of LNAs can be further increased by their ability to get freely incorporated in DNA to form chimeric gapmers, whereby a central DNA portion is flanked on both sides by LNAs. Such chimeric LNA-ODNs allow the improved affinity and higher nuclease resistance of LNA to be combined with the ability of gapmers to recruit RNAse H [14].

## Mechanism of Action of Antisense Oligonucleotides

**Figure 2:**
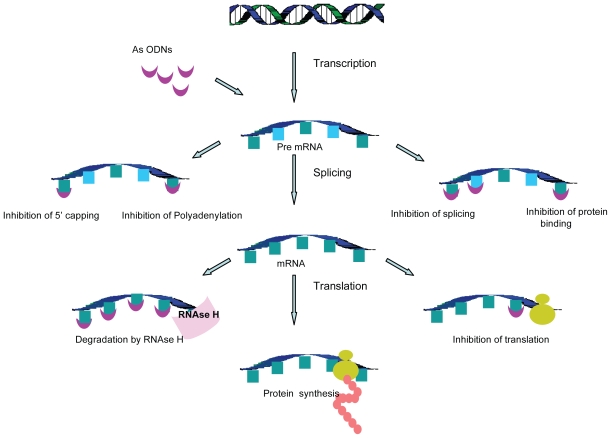
Mechanism of action of antisense oligonucleotides. As-ODN enters the cell and interacts with the target mRNA by sequence-specific base pairing. The As-ODN-mRNA duplex then prevents protein synthesis by interfering with various steps of mRNA synthesis.

As-ODNs are designed to modulate the expression of proteins encoded by the mRNA, by binding and interfering with the function of target mRNA. Prior to protein synthesis, mRNA undergoes a range of essential processing steps such as 5′-capping, polyadenylation, intron—exon splicing, nuclear export, cytoplasmic stabilization and ribosomal binding. All of these steps are crucial for the synthesis and function of mRNA and are highly regulated. By interfering with any one of these processing reactions, As-ODNs can produce their inhibitory effects (Fig. 2).

A most important mechanism employed by As-ODNs to cause the knockdown of proteins is the activation of RNAse H enzyme. RNAse H is a ubiquitously expressed endonuclease that recognizes and hydrolyzes the RNA strand of the mRNA-olignucleotide heteroduplex [15]. The cleavage leaves As-ODNs intact and free to bind to another copy of mRNA. This recycling of As-ODNs makes their effect long lasting, making it possible to use them in micro or nano molar concentrations. As-ODN mediated RNAse H-dependent downregulation of mRNA is quite efficient and can be up to 85%–95% of the control levels. The chemistry of the mRNA-oligonucleotide duplex decides whether or not it will be recognized by RNAse H. While phosphodiester and PS-ODNs can be recognized by RNAse H, the majority of other sugar or backbone modifications render the As-ODN incapable of activating RNAse H. It is also important to note that RNAse H activity is located predominantly in the nucleus and thus the nuclear localization of RNAse-dependent As-ODNs becomes virtually essential for their function [16].

As-ODNs, which are not able to activate RNAse H, produce their effects by targeting other steps of the mRNA synthesis. These other mechanisms of As-ODN inhibition can be classified into either “occupancy only mediated mechanisms” or “occupancy activated destabilization” [17].

“Occupancy only mediated mechanisms” exploit the binding of As-ODNs to their specific sequences in mRNA, thus inhibiting the interaction of mRNA with nucleic acids, proteins and other factors vital for its processing. One such step is the splicing of mRNA that involves the excision of non-protein coding intervening RNA sequences (introns). The splicing reactions are sequence-specific and require the concerted action of spliceosomes. Therefore, the As-ODNs that bind to the sequences required for splicing will either physically prevent the required cleavage reactions or will prevent the binding of necessary factors. This will eventually result in inhibition of the production of the mature protein [17]. The modulation of splicing by As-ODNs can also be used for the correction of aberrant splicing and restoration of a functional protein [18].

Another mechanism by which As-ODNs can downregulate expression of mRNA is by inhibition of mRNA translation. As-ODNs directed to the translation initiation codon of mRNA can bind to it, resulting in the blockade of protein translation [19].

“Occupancy activated destabilization” methods target the steps of mRNA synthesis that are essential for its stabilization, such as 5′ capping or 3′ polyadenylation of mRNA. 5′ capping is crucial not only for the stability of mRNA, but also plays a role in its binding to the nuclear matrix and its transport out of the nucleus. Likewise, polyadenylation is essential for the stability and transport of mRNA. Therefore, As-ODNs designed against the 5′ or 3′ region of pre-mRNA can prevent its capping or Polyadenylation, resulting in the destabilization and disintegration of mRNA [17].

## Delivery of Antisense Oligonucleotides: In Vitro and in Vivo

As-ODNs enter the cells mainly by endocytosis [20], caveolar potocytosis [21] or by pinocytosis [22] and accumulate in the endosomal/lysosomal compartment. Most of the As-ODN is degraded there and only the small portion that escapes to the cytoplasm and the nucleus is responsible for its pharmacological effects [20, 21].

In order to produce their biological effects, it is crucial for As-ODNs to be able to effectively penetrate the target cells. However, various biological barriers hinder the transport of As-ODNs to the target site. The large size and ionic charges of As-ODNs makes it relatively difficult to cross the plasma membrane. Moreover, As-ODNs need to cross the endosomal/lysosomal barrier and escape degradation by nucleases, in order to reach the target site, otherwise their concentration at the target site may become too low to produce any significant biological effect [23]. Lastly, for effective systemic therapy, the integrity of As-ODNs must be maintained in the blood for a sufficient length of time to allow As-ODNs to reach their target sites [24]. Therefore, as discussed earlier, modifications have been made in As-ODN structure to improve their bioavailability. In addition, a large variety of delivery systems have been developed to enhance the cellular uptake of As-ODNs, to protect them from degradation and to improve their intracellular, and especially, intranuclear delivery.

ODN-lipid conjugates or liposome formulations are most commonly used as As-ODNs carriers in vitro. Cationic liposomes, such as Lipofectin and Transfectam, encapsulate As-ODNs to protect them from nuclease degradation and neutralize their negative charge to ease their entry into the cell: the addition of fusogenic lipids, such as dioleyl phosphatidylethanolamine (DOPE), to liposome formulations aids in destabilizing the endosomal membrane, thus facilitating the delivery of As-ODNs to the target site [25–27]. However, cationic liposomes exhibit a markedly decreased activity in the presence of serum and antibiotics. Moreover, their utility is limited in vivo because of their toxicity and serum sensitivity [28].

Carrier molecules, that utilize receptor mediated endocytosis (RME), make use of import mechanisms already present in the cell membrane for the transport of essential nutrients. As-ODNs are linked to the carrier proteins, either by covalent bond, or can be linked non-covalently via poly-l-lysine-carrier conjugates. The choice of the carrier depends on its ability to bind to a certain cell surface receptor, allowing As-ODNs to be delivered effectively to target cells that express or over-express that particular receptor [29, 30]. Covalent conjugation of As-ODNs to macromolecules, like dendimers, has also been shown to enhance the uptake and retention of As-ODNs in the cells. Unlike liposome formulations, the complexes of As-ODNs and dendimers are stable in the presence of serum [31, 32]. Peptides, such as fusogenic peptides that aid in the fusion of ODN—peptide conjugates with the cellular membranes, signal import peptides that improve the cellular uptake of As-ODNs or nuclear localization signal (NLS) peptides that help in targeting the ODNs to the nucleus, have also shown to be effective in in-vitro studies [33–35]. Covalent coupling of As-ODNs to these peptides allows them to enter the cells by receptor and transporter independent mechanisms, thus enhancing their penetration into the cells. Biodegradable nanoparticles have also been investigated as delivery vehicles for As-ODNs. ODNs are adsorbed to the surface of nanoparticles by hydrophobic interactions. To promote the binding between As-ODNs and nanoparticles, hydrophobic cations, such as quarternary ammonium salts, are usually used. Although in vitro studies have shown nanoparticles to be effective carriers of As-ODNs [36], little is known about their in vivo efficacy.

Another approach to enhance As-ODN internalization into the cells is to generate transient permeabilization of the plasma membrane, thus allowing As-ODNs to enter the cells by diffusion. Transitory pores are formed in the cell membrane, either chemically by streptolysin O [37], or mechanically by electroporation [38], shockwave or ultrasound waves [39].

Surprisingly, though delivery vehicles are essential for the uptake and efficacy of As-ODNs *in vitro*, most of the As-ODN drugs in clinical trials are being administered as saline solutions without delivery vehicles. While it is possible that As-ODN uptake *in vitro* might be different from *in vivo* uptake and endosomal/lysosomal sequestration might not be a problem *in vivo*, the widely accepted dogma is that the presence of “endogenous biological carriers” facilitates the physiological delivery of As-ODNs to the targeted site. However, despite numerous reports of As-ODNs binding to plasma proteins *in vivo*, no specific carrier has yet been identified [40].

## Pharmacokinetics of Antisense Oligonucleotides

The studies investigating the pharmacokinetic properties of As-ODNs have used various routes of administration; including subcutaneous (s.c.), intradermal (i.d.), intraperitoneal (i.p.) and intravenous (i.v.) and topical applications. The pharmacokinetic profile of As-ODNs, though independent of their length and specific sequence, is influenced by their chemical composition.

PS-ODNs readily bind serum proteins, especially albumin and α-2 macroglobulin, which prevents rapid clearance by glomerular filtration, thus prolonging the plasma half life of these drugs and providing them the opportunity to be distributed to the peripheral tissues [41]. At clinically relevant doses, more than 90% of PS-ODN is bound to plasma proteins [42]. The binding affinity for serum proteins is species dependent and follows this order; guinea pig > rat > rabbit > human [40]. However, the serum protein binding is saturable, resulting in excretion of intact oligomers at higher doses [43].

There is rapid clearance of PS-ODNs, following i.v. administration, with a plasma half life of less than an hour and the concentrations becoming undetectable within 5–6 hours. The assays performed with radio labelled ODNs show that the plasma clearance is bi-phasic and the terminal elimination half life is around 40–60 hours [43].

In the body, the PS-ODNs are widely distributed to the tissues, with the peak concentrations in highly perfused organs such as liver, kidney and spleen [44]. The tissue half life of PS-ODNs is much longer, ranging from one to five days, though only barely detectable levels of full length ODNs are present in tissues after 48 hours [45]. PS-ODNs are distributed not only to the tissues but are also able to penetrate the cells in the tissues. Even though at early time points after PS-ODN injection most of it is associated with extracellular matrix and connective tissue, by 24 hours almost all of it is found within the cells [46].

The metabolism of PS-ODNs, primarily mediated by endo and exo nucleases, results in shorter nucleotides, and ultimately nucleosides, that are degraded by normal metabolic pathways. The elimination of PS-ODNs is mainly through urine [47] and to a lesser extent through the faeces.

The pharmacokinetics of second and third generation As-ODNs has also been investigated. The second generation As-ODNs have a rapid plasma distribution phase of 4–6 hours, followed by a slower distribution phase [48]. The second generation ODNs are rapidly distributed to the tissues, with the highest concentration in liver, kidney and spleen, and have a tissue half life of 22 days [49]. The third generation As-ODNs, such as MFs, also exhibit a favorable pharmacokinetic profile. They have high aqueous solubility and stability [50]. Systemic administration of MFs is followed by a rapid distribution phase of 1–4 hours and a plasma half life of 1–9 hours. The accumulation is rapid in the various organs, especially in the liver and kidney. Elimination is mainly by urine and faeces [51].

## Potential Side Effects of Antisense Oligonucleotides

A number of studies have extensively studied the toxicological profile of As-ODNs in various species including mice, rats, monkeys and humans and show that all three generations of As-ODNs have an acceptable safety profile.

Studies of the most commonly used As-ODNs, PS-ODNs, show that the modifications in backbone result in non-specific protein interactions. This is because, as PS-ODNS are more negatively charged as compared to phosphodiester ODNs, their polyanionic nature enhances their affinity for proteins, such as heparin-binding proteins, cell surface proteins, viral protein CD4, HIV glycoprotein 120, epidermal growth factor receptor (EGFR), platelet-derived growth factor (PDGF) and certain isoforms of protein kinase C (PKC), resulting in non-specific side effects. For example, PS-ODNs can induce autophosphorylation of EGFR and thus, prevent the binding of EGF to its receptor [52].

Most commonly reported serious dose limiting acute toxicities of As-ODN administration are: transient activation of the complement cascade, prolongation of partial thromboplastin time (PTT), thrombocytopenia and elevation of serum transaminases. Most of these toxicities are the result of non-specific interactions between As-ODNs and plasma proteins. For example, PS-ODNs may interact with factor H, a circulating negative regulatory factor, leading to activation of the complement cascade via an alternative pathway, resulting in increased complement split products, such as C3a and C5a, and subsequent cardiovascular events, such as hypotension. The altered clotting profile can be due to PS-ODNs binding to multiple coagulation factors, such as VIIIa, IXa, X and II, leading to a transient self-limited prolongation of activated partial thromboplastin times [53]. Moreover, PS-ODNs with four or more contiguous guanosine residues can form quadruple-stranded tetraplexes and other higher-order structures, where each guanosine residue is hydrogen bonded to another guanosine in the quartet. These highly negatively charged molecules can be extremely biologically active, resulting in non-specific side effects, such as inhibition of smooth muscle proliferation or induction of bone marrow macrophage progenitor cells proliferation [54, 55].

As-ODNs containing CpG motifs result in stimulation of the immune response in mammalian systems. This is because CG dinucleotide is more frequently found in viral and bacterial DNA than in the human genome and is usually present in the methylated form in the vertebrate system. Therefore, unmethylated CpG motifs normally act as a marker for the immune system to signify infection [56]. Various types of immune cells have pattern recognition receptors that can recognize unmethylated CpG dinucleotides, resulting in stimulation of immune cells [57]. Unmethylated CpG dinucleotides produce immune responses similar to T-helper type 1 (TH_1_)-cell responses, resulting in the activation of natural killer (NK) cells, dendritic cells, macrophages and B cells [58]. The stimulation of the immune system by CpG dinucleotides can result in splenomegaly, lymphoid hyperplasia and diffused infiltration of mixed mononuclear cells in multiple organs [59].

A lot of attention has been paid to genotoxicity of As-ODNs. This is because theoretically, there is risk of As-ODNs getting integrated into the human genome, resulting in mutagenesis. In addition, there is the risk of ODNs being degraded to carcinogenic or mutagenic metabolites [60]. However, studies of As-ODNs in primates and rodents have shown no evidence of teratogenic effects or changes in reproductive performance or fertility [61].

It is important to note that As ODNs are generally safe at therapeutic doses. The As-ODN induced toxicities occur at a dose exceeding the therapeutic dose normally used in the clinical trials [59]. Moreover, modifications in the chemistry of As-ODNs have resulted in safer toxicity profiles, with lesser stimulation of the immune system and fewer non-specific effects as compared to first generation As-ODNs [62].

## Antisense Oligonucleotides in Therapeutic Intervention

The first drug trial for As-ODNs, conducted in 1992 for the treatment of leukemia [63], fuelled optimism about the therapeutic potential of As technology. However, a mounting amount of evidence showed there were non-specific effects of As-ODNs, creating doubts about the clinical suitability of As-ODN drugs. After numerous clinical trials, Vitravene (ISIS Pharmaceuticals), developed for the treatment of cytomegalovirus (CMV) induced retinitis in AIDS patients, became the first drug to get United States Food and Drug Administration (FDA) approval. The past decade has seen an exponential increase in the use of As-ODNs in various in vitro and in vivo models, where As-ODNs are being explored as potential therapeutics against cancer, viral infections, and inflammatory diseases, among a multitude of other diseases. More importantly, the effectiveness of more than 30 therapeutic As-ODNs is currently being tested in clinical trials some of which are listed in Table 1. The growing optimism about As therapeutics is due to the certain advantages this technology has over traditional therapeutic interventions, some of which are discussed below.

### Rational drug design

Rational drug design for any disease requires the identification of an appropriate target and the development of a drug with a specific affinity to that target. In contrast to some drugs, where the mechanism of action is not well understood, As therapeutics is based on the well defined principle of Watson Crick hybridization and the ability of As-ODNs to bind the target mRNA in a sequence specific manner resulting in the knockdown of target gene. Thus, the use of As-ODNs permits the treatment of almost any disease that is linked to a dysregulated gene expression.

### Easy synthesis

The ease of synthesis of As-ODNs is by virtue of the fact that the only information that is required for synthesis is the target mRNA sequence. From the first use of manually synthesized ODNs in 1977, As-ODN technology has progressed to a stage where automated synthesis of oligonucleotides can be commercially done on a mass scale in weeks. Various computational algorithms that aid in designing potent As-ODNs are commercially available. These include oligonucleotides array, RNAse H mapping and mRNA walking. The *m*fold and *s*fold programs available in the public domain are also widely used for predicting the structure of the target mRNA sequence [53].

The progress in the use of As-ODN technology has been further facilitated by the advances in the field of nucleic acid chemistry. Now it has become easy to synthesize a stable complementary ODN sequence, that will hybridize to the sense strand with high affinity and inhibit its function, as long as the sequence of the gene and consequently the target mRNA is known.

### Specificity

The specificity of Watson-Crick hybridization between As-ODN and mRNA suggests that the use of As-ODNs will result in sequence specific downregulation of protein. The use of As-ODNs permits the knockdown of a specific target protein, from a group of closely related proteins, at the mRNA level.

In addition, as the pattern of gene expression varies between normal cells and diseased cells, such as tumour cells, As-ODNs can be used specifically against mutated or tumour associated genes without affecting the expression of proteins in normal cells.

### Accessibility

As opposed to the conventional treatment, directed at a wide range of cellular targets, the more recent focus in drug development has shifted mainly to inhibition of individual proteins. However, because of their intracellular localization, not all proteins are accessible to small molecules (e.g. inhibitors of cell signalling proteins such as kinases) or macromolecular antibodies, whose use is mostly limited to cell surface receptors and secreted proteins, due to their inefficient delivery into the cells. As-ODNs, on the other hand, offer the opportunity to target those proteins, at the gene expression level in vivo, that can not be easily targeted by other available pharmacological tools [64].

### Ease of translation/direct clinical application

Another advantage of As technology is that the oligonucleotides that have been used for inhibition of gene expression in the cell culture system and have shown therapeutic benefit in the treatment of a disease, can be used directly in animal models of the disease and for the disease treatment in humans. Thus, the use of As-ODNs is advantageous, as opposed to some of the traditional target validation approaches, that might be useful in preclinical models for target identification but are unsuitable for human use. In fact many of the As-ODN drugs that are currently in clinical trials were used initially for target identification in in vitro systems.

## Antisense Oligonucleotides in Cancer Therapeutics

Since the first trial to knock down N-Myc protein in vivo [65], tremendous progress has been made in the field of oncology with encouraging results. The main focus is on the knockdown of the genes that become upregulated during tumorogenesis such as the genes involved in apoptosis, cell growth and survival, angiogenesis and metastasis.

Of these, the most promising candidates for antisense therapy are those molecules that have been shown to be causally related to cancer progression or therapeutic resistance and are not amenable to inhibition by the conventional therapy [66]. Some of the main targets of As-ODNs in cancer therapeutics are discussed below.

### Bcl-2

Bcl-2 is a mitochondrial-membrane protein with the ability to block the apoptosis. Over-expression of Bcl-2 has long been linked to tumorogenesis and chemoresistance, suggesting that targeting this gene can enhance chemotherapy-induced apoptosis [67]. Pre-clinical, in vitro and in vivo, studies have shown that anti-Bcl-2 As-ODNs can induce apoptosis in cells derived from solid tumours and sensitize tumour cells to the effect of chemotherapy [68, 69]. G3139 (Oblimersen, also called Genasense; Genta Inc.) is an 18 base PS-ODN targeting the first six codons of the Bcl-2 mRNA open reading frame [70]. Numerous clinical trials have employed G3139 to treat a variety of cancers, such as Multiple Myeloma, Malignant Melanoma, Chronic Lymphocytic Leukaemia (CLL), Non-Hodgkin’s Lymphoma (NHL), breast cancer and Small Cell Lung Cancer (SCLC). The results of several phase I and phase I/II trials of G3139 have been reported [71, 72], showing that Bcl-2 As therapy resulted in the specific downregulation of Bcl-2, without significant toxicity at clinically relevant doses, and therefore has potential for the treatment of cancers, which have Bcl-2 over-expression, resistant to conventional therapy. The drug is currently in phase II and II/III of clinical trials, either alone or in combination with chemotherapy, for a variety of cancers. Recently published results of a randomized phase III trial in patients with metastatic melanoma, comparing the treatment with a combination of dacarbazine and G3139 to dacarbazine alone, have failed to reveal clinically meaningful results [73]. However, in the group of patients who achieved antitumour response, use of G3139 and dacarbazine combination therapy produced a significant increase in progression-free survival, as compared to patients receiving dacarbazine alone. Results from other clinical trials will help to determine the efficacy of this drug.

### Protein kinase C α

Protein kinase C α(PKCα) is a member of family of the cytoplasmic serine-threonine protein kinases involved in a myriad of signal transduction pathways controlling cell proliferation. There are 13 isoforms of PKC and currently available inhibitors and modulators of PKC activity are limited by their inability to differentiate between the various isozymes. Therefore, several As-ODNs, which specifically target individual members of the PKC family, are being used to study the various isoforms of this enzyme. Over-expression of PKCα has been implicated in oncogenesis and tumour progression [74]. Several in vitro and in vivo studies have shown that As-ODNs targeted against PKCα can inhibit tumour growth [75–77]. The efficacy and safety profiles of these results encouraged the human trials of PKCα-specific 20 mer PS-ODN, ISIS 3521 (also referred to as Affinitak, Aprinocarsen and LY90003; Isis Pharmaceuticals), complementary to the 3′-untranslated region of PKCα mRNA. The phase I trials of ISIS 3521 were carried out successfully in patients with treatment-resistant solid tumours [78–80]. The therapy was well tolerated and showed benefit in some cases. Dose-limiting toxicities, moderate fatigue and thrombocytopenia, were encountered at doses of 3 mg/kg per day. Therefore, the dose for Phase II trial was set at 2 mg/kg/day, which corresponded with doses showing antitumour activity in xenograft models. Phase I/II studies, evaluating the combination therapy of ISIS 3521 and chemotherapeutic agents, were initiated in patients with stage III B or IV NSCLC [81]. The drug combinations were well tolerated with acceptable toxicity profiles. Based on phase II, results two phase III randomized trials were initiated in patients with NSCLC. The results failed to show any benefit in terms of median survival rate in the group receiving ISIS 3521 (ISIS Pharmaceuticals). Disappointing results have also been shown in a phase II trial of ISIS 3521, in patients with metastatic colorectal cancer, with very little uptake of the drug in tumour cells and no evidence of significant knockdown of PKCα expression [82]. More studies need to be done to evaluate the efficacy of this drug.

### H-ras

The proteins encoded by members of the ras family serve as critical components of the cell-signaling pathway and are involved in the control of cellular proliferation, differentiation, and cell death. Mutations in the genes encoding the Ras family of proteins result in abnormal cell growth and malignant transformation. Activating mutations in the ras family of genes are found in over 30% of human tumours [83], making ras a promising therapeutic target. A 20 mer PS-ODN, ISIS 2503 (ISIS Pharmaceuticals), targeted to the translation initiation region of H-ras mRNA selectively reduced the expression of H-ras protein in vitro [84]. In various phase I clinical trials ISIS 2503 did not show any dose limiting toxicity. However, no consistent decrease in H-ras mRNA, levels, in peripheral blood lymphocytes, was observed [85].

A combination of ISIS 2503 and gemcitabine, in metastatic or locally advanced pancreatic adenocarcinoma, showed a promising response rate, but no clear deductions could be made about the overall benefit for this combination therapy, warranting further evaluation of the efficacy of ISIS 2503 [86].

### c-raf

Raf kinases, members of the family of serine/threonine protein kinases, are downstream mediators of mitotic signalling pathways. Most notably, raf-1 plays a central role in the mitogen activated protein kinase (MAPK) pathway and is activated by ras protein [87]. Raf has also been reported to bind to Bcl-2 and to be indirectly involved in the regulation of apoptosis [88]. Mutations of the raf proteins, resulting in their constitutive activation, have been identified in many tumours [89]. Therefore, targeting raf seems to be an attractive option for the treatment of diseases associated with abnormal cell proliferation. In a screening study comprising 34 different As-ODN sequences, a 20 mer PS-ODN (ISIS 5132; ISIS Pharmaceuticals), directed to the 3′ untranslated region of the c-raf mRNA, resulted in a most potent inhibition of the growth of human tumour cell lines in vitro and in vivo [90]. ISIS 5132 was shown not only to decrease c-raf expression but also to increase tumour sensitivity [91]. In a phase I trial, changes in the expression of c-raf1 mRNA were assessed in peripheral blood mononuclear cells, collected from patients with advanced cancers treated with ISIS 5132. Significant reductions from baseline were detected in the expression of c-raf1 [92]. Results of other phase I clinical trials of ISIS 5132, conducted in patients with a variety of advanced solid tumours, showed that the drug is generally well tolerated with only mild side effects, which were generally the same as those attributed to PS-ODN treatment. Even though no evidence of significant tumour regression was observed in these trials, several of the patients experienced long periods of disease stabilization [93–95]. The antitumour activity of ISIS 5132 has been evaluated in phase II studies in patients with colorectal cancer, ovarian cancer, hormone refractory prostate cancer, small cell and non small cell lung cancers [96–99]. Again no clinically significant tumour regressions were observed, but protracted periods of stable disease were observed in some patients.

Another raf-1 As-ODN, LErafAON (NeoPharm), a 15-mer As-ODN directed to the translation initiation site of c-raf-1 mRNA, has also entered clinical trials. The PS modification of this As-ODN is limited only to the terminal base at the 3′ and 5′end [100]. LErafAON has been encapsulated in a cationic liposome in order to protect the ODNs from degradation and to improve their serum half life. Preclinical studies of this drug have shown inhibition of tumour growth, more than 50% inhibition of raf-1 expression in tumour xenografts and increased sensitization of tumour cells to radiation and to chemotherapeutic agents [101, 102]. Phase I study of LErafAON has shown that the i.v. delivery of drug is well tolerated along with palliative radiotherapy [103]. A phase I dose-escalation trial, using a weekly bolus regimen of LErafAON in patients with advanced solid tumours, has shown dose-independent hypersensitivity reactions and dose-dependent thrombocytopenia, limiting tolerance of the drug [104]. Another phase I study of combined modality treatment using LErafAON and radiation therapy, in patients with advanced tumours, has shown that the combination is well tolerated. However, pre-medication with steroids and antihistamines was needed to control the infusion related reactions. Therefore, modifications are being made in liposomal formulation to improve the tolerability of LErafAON [105].

## Antisense Oligonucleotides in Viral Therapeutics

A lot of attention is currently being paid to the treatment of viral cytopathic effects by As-ODNs. Many valuable contributions have been made to the As-ODN antiviral strategies by studies involving various viruses, such as Herpes Simplex virus (HSV), Human Immunodeficiency Virus (HIV), Cytomegalovirus (CMV), Hepatitis B virus (HBV), Hepatitis C virus (HCV), Epstein—Barr Virus (EBV) and many more. Some of the antiviral As-ODNs are discussed below.

### Cytomegalovirus (CMV)

Cytomegalovirus (CMV) belongs to the Herpes-viridae family of viruses, which are DNA viruses that exhibit the biological properties of latency and reactivation. In the developed countries, up to 80% of individuals develop sub-clinical CMV infections. In most of the cases, the primary CMV infections go unrecognized, however, in immunocompromised patients, CMV infections are one of the major causes of morbidity and mortality [106]. CMV retinitis is one of the most common opportunistic infections in patients with acquired immunodeficiency syndrome (AIDS). AIDS patients infected with CMV retinitis can develop either intolerance or resistance to commonly used anti-CMV treatment regimens, necessitating the development of alternative treatment options [107]. One such option is the use of As-ODNs for the inhibition of CMV replication. Much of the research on inhibition of CMV replication by As-ODNs has mainly focused on inhibition of CMV immediate-early (IE) gene products. In one of the early studies, PS-ODNs complementary to the mRNA of the CMV DNA polymerase gene or to RNA transcripts of the major immediate-early regions 1 and 2 (IE1 and IE2), showed antiviral activity. ISIS 2922 (Fomivirsen also called Vitravene ; ISIS Pharmaceuticals), a PS-ODN with a 21 nucleotide sequence complementary to RNA of IE2, showed at least 30 fold more potent antiviral activity as compared to nucleoside analog gangci-clovir. The results showed that ISIS 2922 inhibits viral production in a specific and dose dependent manner [108]. Subsequent reports showed that antiviral activity of ISIS 2922 is because of anti-sense, non-antisense sequence dependent and sequence independent mechanisms. Base complementarity to target RNA was important for optimal activity of ISIS 2922 in antiviral assays, but base changes affecting parameters other than hybridization affinity also influenced antiviral activity. Moreover, the drug also caused sequence-independent inhibition of virus adsorption to host cells at high concentrations [109]. Following encouraging data about the antiviral activities of ISIS 2922, clinical trials of the drug were carried out for the treatment of CMV retinitis in AIDS patients. The results of phase I, II and III trials showed intravitreal injections of ISIS 2922 to be safe and effective. The drug halted the progression of both acute and chronic CMV retinitis in AIDS patients. Moreover, the local treatment with the drug reduced the incidence of systemic side effects. Most commonly reported side effects were increase in intraocular pressure and mild to moderate intraocular inflammation, both of which were transient or treatable with topical steroid treatment [110, 111]. Following the successful clinical trials, in 1998, ISIS 2922 was approved by FDA, for the treatment of CMV-induced retinitis in patients with AIDS and became the first As-ODN drug to be marketed commercially [112].

### Human immunodeficiency virus (HIV)

HIV is a single stranded RNA virus that uses reverse transcriptase (RT) to create a DNA copy of its RNA genome. The viral DNA then gets integrated into the DNA of the host, and is subsequently transcribed and translated by the host cell. HIV infection is spreading worldwide at an alarming rate, representing the difficulties in controlling viral replication. Many regions of the HIV genome have been targeted with As-ODNs, including the rev, tat, gag, pol, and env genes, 5′ untranslated region and psi sequences. Various ODNs have been shown to be effective in acute and chronic infections [113–115]. GEM 91 (Hybridon), a 25-mer PS-ODN that binds to the translation initiation site of the HIV gag mRNA, was shown to effectively reduce HIV replication in vitro [116]. Although the initial reports of GEM 91 clinical trials showed it to be well tolerated [117], its use was later discontinued because of dose-limiting thrombocytopenia and elevated serum transaminase levels. GEM 92 is an orally administered second generation As-ODN synthesized on a truncated GEM 91 sequence [118]. GEM 92 is currently in phase I clinical trials, with preclinical studies showing an improved stability and safety profile [118].

### Hepatitis C virus

Chronic infection by HCV is the most common cause of hepatocellular carcinoma, and is the primary reason for liver transplantations among adults in the western world. However, currently available anti-HCV compounds are not broadly effective, especially against the chronic HCV infection. Therefore, continuous efforts are being made to develop newer and better therapeutic strategies [119]. Recently, much attention is being paid to the use of As-ODNs for the treatment of HCV infection. Several As-ODNs that have been designed to bind to the stem-loop structures in the HCV Internal Ribosome Entry Sites (IRES) have been shown to be effective in inhibiting HCV replication in cell-culture assays [120, 121]. The expression of HCV luciferase reporter gene was shown to be inhibited significantly by PS-ODN in the livers of mice infected with recombinant vaccinia virus expressing the reporter construct [122]. ISIS 14803 (ISIS Pharmaceuticals), a 20-nucleotide, 5′-methylcytidine PS-ODN, is being evaluated in phase I and II of clinical trials involving patients with chronic HCV infections [123]. The compound administered, intravenously or subcutaneously, 3 times weekly did demonstrate a significant decrease in HCV RNA levels for a small sub-group of participants, however: many of the patients experienced frequent elevations in hepatic transaminase levels, raising doubts about the suitability of this drug [124].

## Antisense Oligonucleotides in Allergic, Inflammatory and Autoimmune Diseases

As-ODNs have been explored for their therapeutic benefits in various allergic and inflammatory diseases e.g. in bronchial asthma, Crohn’s disease (CD), Ulcerative colitis (UC), psoriasis. Some of the important targets of As-ODN therapy for these disease models are discussed below.

### Intercellular adhesion molecule-1 (ICAM-1)

ICAM-1 is a transmembrane glycoprotein that is constitutively expressed at low levels by vascular and colonic endothelium and a subset of leucocytes [125]. Surface expression of ICAM-1 is increased in response to various inflammatory stimuli, resulting in the adhesion and recruitment of monocytes, macrophages, neutrophils, T lymphocytes and dendritic cells (DCs) to the site of inflammation [126]. Thus, theoretically, it seems likely that inhibiting the expression of ICAM-1 by As-ODNs would hinder the migration of immune cells, resulting in a potent immunosuppressive effect. Results from pre-clinical studies showed that using As-ODNs targeted to ICAM-1 results in a decreased receptor expression and reduction in inflammation [127]. ISIS 2302 (Alicaforsen; ISIS Pharmaceuticals) is a 20 base PS-ODN that binds to the 3′-untranslated region of the mRNA and prevents further translation, resulting in knockdown of ICAM-1 expression [128]. Several placebo-controlled randomized trials with an ISIS 2302 have been performed in active steroid-treated Crohn’s Disease (CD). The pilot phase I study involving 20 participants showed significant therapeutic benefit in chronically active CD [129]. The initial enthusiasm was, however, dampened by the two follow-up large multicenter phase II trials, using subcutaneous and i.v. formulations of the drug, which showed no significant difference between the placebo and treatment arms [130, 131]. On the other hand, ISIS 2302 enema formulation has shown significant acute and long term benefits with favourable safety profile in Phase I and II trials, in patients with mild to moderate descending ulcerative colitis (UC) and chronic pouchitis [132, 133]. Following these encouraging reports, further trials using ISIS 2302 enema were carried out in patients with UC. The results of these trials suggest a more long term reduction in disease activity in patients receiving ISIS 2302 enema, compared to patients receiving placebo or mesalazine, used normally for the treatment of mild-moderate left sided UC [134, 135]. Another recently published phase I/II study has shown that ISIS 2302 enema is useful for the treatment of local inflammation in patients with active UC and has low systemic bioavailability and exposure [136].

ISIS 2302 has also been evaluated in combination with a cyclosporine-prednisone regimen in phase I and II trials for the prophylaxis of acute allograft rejection [137]. Even though ISIS 2302 was well tolerated, no significant differences were noted, in terms of acute graft rejection or improved graft survival, among the patients receiving cyclosporine-prednisone alone or in combination with ISIS 2302. Further evaluation of ISIS 2302 needs to be done to determine its usefulness for the treatment of various inflammatory conditions.

## Tumour Necrosis Factor-α (TNFα)

TNFα is a potent pleiotropic cytokine that is produced by most cells of the body but especially by cells of monocyte lineage. TNFα plays an important role in the initiation of a vigorous cell and antibody mediated immune response and in expansion of the required lymphocyte populations [138]. Therefore, it is not surprising that targeting TNFα has been the focus of for the treatment of rheumatoid arthritis and other inflammatory conditions. ISIS 104838 (ISIS Pharmaceuticals), a 20 nucleotide gapmer targeting the mRNA of human TNFα, is the first As-ODN belonging to the second generation to be tried in clinical trials [139]. The preclinical studies with ISIS 104838 showed it to be effective for the treatment of collagen induced arthritis with activity comparable to that by anti-TNFα antibody [140], and that it successfully knocks down TNFα expression by 85% in stimulated keratinocytes [48]. In phase I of clinical trials, ISIS 104838 was administered either by i.v. or s.c routes [48]. The drug was well tolerated, the principle side effects being redness at the site of injection and prolongation of PTT that was transient and was not associated with bleeding. As expected of a second generation As-ODN, ISIS 104838 had a longer plasma half life, with a terminal plasma half life of two weeks. Moreover, in the blood obtained from study subjects and then stimulated with LPS, ex vivo production of TNFα was significantly decreased in a dose dependent manner. ISIS 104838 is currently in phase II of clinical trials for the treatment of rheumatoid arthritis (RA) and CD.

## Adenosine Receptor-1 (AR-1)

Adenosine is a ubiquitous purine nucleoside, normally present at low concentrations in the extracellular space, but its levels are greatly increased under metabolically stressful conditions. An increasing amount of evidence suggests that adenosine is a pro-inflammatory mediator that induces bronchoconstriction in animal models and in patients with inflammatory airway diseases, such as asthma and chronic obstructive pulmonary diseases (COPD) [141]. Adenosine receptor, AR-1 has been shown to be up-regulated in bronchial smooth-muscles of asthmatic patients, in animal models of the disease [142, 143] and in bronchial smooth-muscle tissue exposed to human asthmatic serum [144]. Respirable As-ODNs (RASONs), as compared to systemically administered As-ODNs, offer the potential to selectively downregulate the AR-1 expression in the lung at much smaller doses and also minimize the risk of systemic side effects and toxicity. Moreover, lungs are lined with surfactant that contains cationic lipids, resulting in enhanced uptake of As-ODNs [145]. EPI 2010 is a RASON designed to target the initiation codon of the human A_1_ receptor mRNA [146]. The pre clinical studies with this drug have shown it to be specific, effective and safe [147, 148]. EPI 2010 was able to block airway hyper-responsiveness (AHR) to inhaled adenosine for several days. Therefore, once weekly administration was selected for the treatment of asthma during phase I clinical trials. Results of Phase I/IIa clinical trials have shown EPI-2010 to be safe and well-tolerated, with modest indications of efficacy in patients with mild asthma [149]. Further clinical trials are ongoing to determine its clinical efficacy.

## Antisense Oligonucleotides in Target Indentification/Validation

The past two decades have seen a dramatic shift in the way the pharmaceutical companies approach the development of new drugs. Traditionally, the compounds were investigated for their biological effects first, such as the effect of anticancer agents on cell proliferation, and were sometimes marketed even before their exact mode of action was known. A classic example of this is the commercial use of aspirin long before its biological target was identified. This approach, though successful in some cases, also resulted in many failures due to various factors such as, insufficient specificity of the compound, unsuitability of the target etc [150]. Consequently, the pharmaceutical companies suffered enormous losses. To circumvent these problems, many companies have now adopted a “target directed” approach. The main goal is to understand the cellular mechanisms responsible for various diseases and to investigate if downregulating a certain target protein would produce the desired biological effects. The information thus obtained is then used to develop new drugs [150].

It had always been a challenge to elucidate the function of only one particular isoform of a protein, while leaving the other isoforms intact. Moreover, the use of various small molecule inhibitors, such as kinase inhibitors, for target identification/validation was confounded by the problems of specificity. For example, SB203580 and PD 98059, in addition to inhibiting p38 and p44/42 MAPKs, have been shown to inhibit the activity of cyclooxygenases and thromboxane synthase [151]. With the availability of As-ODNs it has now become possible to dissect complex intracellular signalling cascades with various overlapping pathways and to discern the function of homologous gene products selectively and efficiently. In addition, the As-ODN knockdown of cellular proteins makes it possible to investigate their specific roles in disease development and progression, and to determine if suppressing the activity of that particular protein will significantly influence disease pathophysiology.

After a suitable target has been identified in vitro, its validity needs to be confirmed by in vivo evaluation. Knockout gene models have traditionally been used as the gold standard for determining the gene functions in vivo. However, their use is limited by the length of time required to create and characterize the mutant strain. Moreover, mutation of certain genes is embryonically lethal making it impossible to study them in a knockout animal model. The use of As-ODNs permits the study of the effect of knockdown of the target gene on the whole animal in a rapid manner and at different stages of development, ranging from the embryonic stage to the adult animal [152].

One example of the use of As-ODNs in target identification/validation is the identification of Sphingosine Kinase-1 (SPHK-1) as a key player in the pro-inflammatory responses triggered by TNFα in human monocytes [153] and by C5a in human monocyte-derived macrophages [154]. Following numerous reports of the role of sphingolipid metabolite, S1P, in a variety of cellular processes, such as cell differentiation and proliferation, we investigated if decreasing the production of S1P, by knocking down SPHK expression, would affect the immune cells response to inflammatory mediators. We did this by using As-ODNs directed to the two known SPHK isoforms, SPHK-1 and SPHK-2. Our results showed an important role for SPHK-1, but not for SPHK-2, in the cellular processes triggered by C5a and TNFα. Combined with other data, our results suggest that targeting SPHK-1 can be a useful therapeutic strategy for the treatment of various allergic and inflammatory diseases.

## Limitations of Antisense Oligonucleotides

Despite the apparent simplicity of the principle, execution of As therapeutics has been very challenging. The main limitation of As therapy is the single target approach. This is because many diseases are caused by multiple, unrelated genetic alterations. Therefore, knocking down the expression of a single target gene may not be sufficient to influence the disease pathogenesis. In phase II and phase III of clinical trials many As-ODN drugs failed to produce significant therapeutic benefit when used alone for the treatment of cancer. A possible explanation is that the proteins regulating the cell cycle progression and cell proliferation are numerous and redundant. Therefore, even if a single target is significantly knocked down, other proteins of the same family may be activated and compensate for the reduced activity of the knocked down protein. One such example is the recently published results of a phase III trial of the drug, Affinitak, directed against PKCα (ISIS Pharmaceuticals). When used for the treatment of non-small cell lung cancer (NSCLC), the administration of the drug, in combination with chemotherapeutic agents, did not result in overall prolongation of life, as compared to chemotherapy alone. The most likely reason for these rather disappointing results is that, as there are 13 isoforms of PKC in mammalian cells, knockdown of only one isoform may not be sufficient and may result in another isoform taking over its function.

Another problem with antisense knockdown is that even though a major reduction in target mRNAs can be achieved, a complete 100% inhibition of proteins is not possible: this is because the transcription of endogenous copies of proteins cannot be inhibited by As-ODNs. This, though useful for the function of normal proteins, can pose problems in case of mutated proteins.

Finally, despite extensive research to improve their delivery and uptake, the in vivo delivery of ODNs still remains one of the major challenges in the field of As therapeutics. The need for a sustained and effective plasma concentration demands administration of As-ODNs by repeated or continuous i.v. infusions. This not only increases the risk of central venous thrombosis and the chances of line infections, but also affects the quality of life of the patients [5]. Delivery systems that will facilitate the delivery and enhance the sustained release of the As-ODNs are needed if As-ODNs are to be incorporated in the routine treatment of patients in clinics.

## Alternatives to Antisense Oligonucleotides

In recent years there has been a tremendous increase in interest in RNA interference (RNAi), especially small inhibitory RNA (siRNA) and microRNAs (miRNAs) as well as ribozymes.

Ribozymes are RNA molecules that act as enzymes and have the ability of breaking and/or forming covalent bonds with extraordinary specificity, thereby accelerating the spontaneous rates of targeted reactions. Many ribozymes catalyze either their own cleavage or the cleavage of other RNAs, but they have also been found to catalyze the aminotransferase activity of the ribosome [155]. Ribozymes occur naturally, but can also be engineered artificially for expression and targeting of specific sequences. At least six types of ribozymes have been successfully modified for therapeutic and functional genomic applications. Of these, the two most commonly used modifications are “hammerhead ribozymes”, which are around 30 nucleotides long and cleave the substrate RNA at sequence NUH (N-any nucleotide, H-not guanosine) and “hairpin ribozymes” that cleave the target RNA immediately upstream of the sequence GUC. The hairpin ribozymes can also catalyze the ligation reactions [156]. Ribozymes are typically expressed from a plasmid vector transfected into the target cell, but they can also be produced in vitro and administered exogenously. Hammerhead and hairpin ribozymes have been successfully used to downregulate specific cellular and viral targets in vitro [157, 158] and in vivo [159]. The success of preliminary studies encouraged the clinical trials of ribozymes, especially for the treatment of infectious diseases and cancer [160, 161].

RNAi is another method that is being increasingly used for stable inhibition of gene expression. RNAi refers to a group of gene silencing mechanisms in which the terminal effector is a 21–23 bp RNA molecule. One commonly employed RNAi mechanism includes introduction of a long ‘triggering’ dsRNA with 2-nucleotide overhangs at the 3′ terminal end, that is subsequently processed into 21–23 bp small interfering RNAs (siRNA), by an RNaseIII-like enzyme called Dicer. This enzyme also includes a helicase domain, suggesting that unwinding of the triggering dsRNA might be required for the subsequent target recognition event, presumably guided by traditional Watson—Crick base-pairing in the enzyme—substrate [ES] complex. This 21–23 bp siRNA species is then incorporated into an, as yet, crudely defined multi-subunit RNA-induced silencing complex (RISC), which targets the unique cellular RNA transcript for enzymatic degradation. RNA hydrolysis occurs within the region of homology directed by the original siRNA, thereby selectively inhibiting target gene expression. RNA-dependent RNA polymerase (RdRp), present in plants but not in vertebrates, amplifies the production of dsRNAs using the anti-sense strand of siRNA as a template [161]. siRNAs have emerged as potent tools to specifically knockdown the target gene expression in a sequence-specific manner [162]. Intense research efforts are being made to develop siRNAs for therapeutic purposes. Various in vitro and in vivo studies have demonstrated the efficacy of this technology [163, 164] and some of the siRNA drugs are currently being tested in clinical trials [165].

RNAi is related to another gene-silencing mechanism that involves a group of small RNA molecules, known as microRNAs (miRNAs). miRNAs are 21–24 nucleotide (nt) non-coding single strands of RNA that are endogenously expressed and are involved in the regulation of mRNA expression. Short hairpin precursors called pre-miRNAs are processed by Dicer into 21-to 23-nucleotide long, miRNA molecules, which are then incorporated into RISC. miRNAs then either cause the translational repression or degradation of target mRNA depending upon the extent of complementarity between the miRNA and target mRNA. If the target mRNA is perfectly complementary to miRNA, miRNA molecules act in a way similar to siRNAs and induce the cleavage of the target mRNA. miRNAs that contain partially complementary sequences to the 3′ untranslated region of mRNAs inhibit their translation [166, 167]. Since the discovery of first miRNA molecules, lin-4 and let-7 in *C. elegans*, [168, 169] many miRNAs have been identified in most of the organisms such as viruses, plants and vertebrates [170]. However, the precise mechanism of action and function of most of the miRNAs still needs to be further elucidated.

The availability of these alternative approaches for sequence-specific knockdown of RNA holds great promise in both research and therapeutics, but the determining factor in choosing one over the other will depend on the cost of production and the ease of delivery to the target cells. Moreover, the experiences with As-ODN therapeutics will give them an advantage against the relative newness of the alternative strategies [171], that are still at the earlier stages of their development, have little information available about their pharmacokinetics and toxicology and need more evidence of their therapeutic potential in vivo.

## Conclusion and Future Directions

The past two decades have seen an increasing use of As-ODNs for the purpose of target identification/validation and the use of the information, thus obtained, for the development of effective therapeutic interventions. Tremendous progress has been made in the understanding and applications of As-ODNs. Most of the structural motifs in PS-ODNs, that interfere with their antisense effects and are responsible for toxicities, have been delineated. Many desirable properties such as efficient uptake by the cells, stability against nucleases, and a strong affinity for target mRNA have been identified. Effective chemical modifications are likely to avoid the non-antisense effects and further enhance the safety and efficacy of As-ODNs and thus expand their potential clinical applications. However, more work needs to be done to further optimize the stability and bioavailability of As-ODNs. Moreover, proof of principle for the biological activity of As-ODNs is needed in the clinical samples, in order to confirm the successful blockade of target protein expression [5]. The rather disappointing results of some of the recent clinical trials indicate a need to clearly establish the relevance of the target to the patient population being studied, an early determination of optimal biological dose and the rational use of combination strategies for the treatment of the disease [66]. Finally, the clinical end points need to be clearly defined in order to evaluate the efficacy of As-ODNs [172].

## Figures and Tables

**Table 1:** Antisense oligonucleotides in clinical trials.

As ODN	Company	mRNA Target	Chemistry	Delivery	Clinical phase	Study group
G3139 (Oblimersen)	Genta	Bcl-2	PS	Systemic	Phase I,II,III	CLL, Malignant melanoma, Multiple myeloma, NSCLC, AML
RESTEN-MP	AVI	c-myc	MF	Systemic	Phase II	Restenosis
ISIS 2503	Isis	H-ras	PS	Systemic	Phase II	NSCLC, breast, colorectal and pancreatic cancer
LErafAON-ETU	NeoPharm	raf kinase	PS	Systemic	Phase I	Advanced cancer
OGX-011	OncoGenex	Clusterin	Gapmer	Systemic	Phase II	NSCLC, Prostate and breast cancer
ISIS 3521 (Affinitak)	Isis/Eli Lilly	PKCα	PS	Systemic	Phase III	NSCLC
GTI 2040	Lorus	Ribonucleotide reductase	PS	Systemic	Phase II	Renal cancer
LR3001	Genta	c-myb	PS	Systemic	Phase I	CML
GEM 231	Hybridon	PKA	Gapmer	Systemic	Phase I,II	Solid cancers
MG98	Methylgene	DNA methyltransferase	Gapmer	Systemic	Phase II	Head, neck and metastatic renal cancers
AP 12009	Antisense Pharma	TGF-β2	PS	Intra tumoral	Phase II	Glioma, malignant melanoma, pancreatic cancer
ISIS 2302 (Alicaforsen)	Isis	ICAM-1	PS	Systemic/enema	Phase II	Ulcerative colitis
EPI-2010	EpiGenesis	Adenosine A1R	PS	Aerosol	Phase II	Asthma
ISIS 104838	Isis	TNF-α	Gapmer	Subcutaneous	Phase III	Rheumatoid arthritis
GEM 92	Hybridon	HIV gag	Gapmer	Oral	Phase II	HIV

**Abbreviations:** As-ODN: antisense oligonucleotide; PKCα: protein kinase Cα; PKA: protein kinase A; TGF-β2: transforming growth factor β-2; ICAM-1: intracellular adhesion molecule-1; Adenosine A1R: adenosine A1 receptor; TNFα: tumour necrosis factorα; HCV: hepatitis C virus; HIV: human immunodeficiency virus; PS: phosphorothioate; MF: morpholino; CLL: chronic lymphocytic leukemia; NSCLC: non-small cell lung cancer; AML: acute myelogenous leukemia; CML: chronic myelogenous leukemia.

## References

[1] Zhang YC Taylor MM Samson WK Phillips MI Phillips MI 2005 Antisense Inhibition 2nd ed Antisense Therapeutics New Jersey Humana Press

[2] Paterson BM Roberts BE Kuff EL 1977 Structural gene identification and mapping by DNA-mRNA hybrid-arrested cell-free translation Proc. Natl. Acad. Sci. U.S.A 74 10 4370 4 270678 10.1073/pnas.74.10.4370 PMC431943

[3] Zamecnik PC Stephenson ML 1978 Inhibition of Rous sarcoma virus replication and cell transformation by a specific oligodeoxynucleotide Proc. Natl. Acad. Sci. U.S.A 75 1 280 4 75545 10.1073/pnas.75.1.280 PMC411230

[4] Dias N Stein CA 2002 Antisense oligonucleotides: basic concepts and mechanisms Mol. Cancer Ther 1 5 347 55 12489851

[5] Vidal L 2005 Making sense of antisense Eur. J. Cancer 41 18 2812 8 16289851 10.1016/j.ejca.2005.06.029

[6] Chen X 2005 Chemical modification of gene silencing oligonucleotides for drug discovery and development Drug Discov. Today 10 8 587 93 15837602 10.1016/S1359-6446(05)03426-4

[7] Kurreck J 2003 Antisense technologies. Improvement through novel chemical modifications Eur. J. Biochem 270 8 1628 44 12694176 10.1046/j.1432-1033.2003.03555.x

[8] Sproat BS 1989 Highly efficient chemical synthesis of 2′-O-methylolibonucleotides and tetrabiotinylated derivatives; novel probes that are resistant to degradation by RNA or DNA specific nucleases Nucleic Acids Res 17 9 3373 86 2726482 10.1093/nar/17.9.3373 PMC317781

[9] Monia BP 1993 Evaluation of 2′-modified oligonucleotides containing 2′-deoxy gaps as antisense inhibitors of gene expression J. Biol. Chem 268 19 14514 22 8390996

[10] Agrawal S 1997 Mixed-backbone oligonucleotides as second generation antisense oligonucleotides: in vitro and in vivo studies Proc. Natl. Acad. Sci. U.S.A 94 6 2620 5 9122245 10.1073/pnas.94.6.2620 PMC20138

[11] Fluiter K 2003 In vivo tumor growth inhibition and biodistribution studies of locked nucleic acid (LNA) antisense oligonucleotides Nucleic Acids Res 31 3 953 62 12560491 10.1093/nar/gkg185 PMC149205

[12] Sazani P 2002 Systemically delivered antisense oligomers upregulate gene expression in mouse tissues Nat. Biotechnol 20 12 1228 33 12426578 10.1038/nbt759

[13] Devi GR 2005 In vivo bioavailability and pharmacokinetics of a c-MYC antisense phosphorodiamidate morpholino oligomer, AVI-4126, in solid tumors Clin. Cancer Res 11 10 3930 8 15897595 10.1158/1078-0432.CCR-04-2091

[14] Kurreck J 2002 Design of antisense oligonucleotides stabilized by locked nucleic acids Nucleic Acids Res 30 9 1911 8 11972327 10.1093/nar/30.9.1911 PMC113840

[15] Wu H 2004 Determination of the role of the human RNase H1 in the pharmacology of DNA-like antisense drugs J. Biol. Chem 279 17 17181 9 14960586 10.1074/jbc.M311683200

[16] Baker BF Monia BP 1999 Novel mechanisms for antisense-mediated regulation of gene expression Biochim. Biophys. Acta 1489 1 3 18 10806993 10.1016/s0167-4781(99)00146-3

[17] Crooke ST 1999 Molecular mechanisms of action of antisense drugs Biochim. Biophys. Acta 1489 1 31 44 10806995 10.1016/s0167-4781(99)00148-7

[18] Sierakowska H 1996 Repair of thalassemic human beta-globin mRNA in mammalian cells by antisense oligonucleotides Proc. Natl. Acad. Sci. U.S.A 93 23 12840 4 8917506 10.1073/pnas.93.23.12840 PMC24007

[19] Dias N 1999 Antisense PNA tridecamers targeted to the coding region of Ha-ras mRNA arrest polypeptide chain elongation J. Mol. Biol 294 2 403 16 10610767 10.1006/jmbi.1999.3277

[20] Shoji Y 1991 Mechanism of cellular uptake of modified oligodeoxynucleotides containing methylphosphonate linkages Nucleic Acids Res 19 20 5543 50 1658734 10.1093/nar/19.20.5543 PMC328955

[21] Zamecnik P 1994 Electron micrographic studies of transport of oligodeoxynucleotides across eukaryotic cell membranes Proc. Natl. Acad. Sci. U.S.A 91 8 3156 60 8159719 10.1073/pnas.91.8.3156 PMC43534

[22] Stein CA 1993 Dynamics of the internalization of phosphodiester oligodeoxynucleotides in HL60 cells Biochemistry 32 18 4855 61 8490026 10.1021/bi00069a022

[23] Lysik MA Wu-Pong S 2003 Innovations in oligonucleotide drug delivery J. Pharm. Sci 92 8 1559 73 12884243 10.1002/jps.10399 PMC7094321

[24] Lebedeva I 2000 Cellular delivery of antisense oligonucleotides Eur. J. Pharm. Biopharm 50 1 101 19 10840195 10.1016/s0939-6411(00)00088-6

[25] Farhood H 1994 Cationic liposomes for direct gene transfer in therapy of cancer and other diseases Ann N Y Acad Sci 716 23 34 discussion 34–5 8024197 10.1111/j.1749-6632.1994.tb21701.x

[26] Capaccioli S 1993 Cationic lipids improve antisense oligonucleotide uptake and prevent degradation in cultured cells and in human serum Biochem. Biophys. Res. Commun 197 2 818 25 8267621 10.1006/bbrc.1993.2552

[27] Farhood H 1992 Effect of cationic cholesterol derivatives on gene transfer and protein kinase C activity Biochim. Biophys. Acta 1111 2 239 46 1420259 10.1016/0005-2736(92)90316-e

[28] Akhtar S 2000 The delivery of antisense therapeutics Adv. Drug Deliv. Rev 44 1 3 21 11035194 10.1016/s0169-409x(00)00080-6

[29] Rojanasakul Y 1997 Antisense inhibition of silica-induced tumor necrosis factor in alveolar macrophages J. Biol. Chem 272 7 3910 4 9020093 10.1074/jbc.272.7.3910

[30] Ginobbi P 1997 Folic acid-polylysine carrier improves efficacy of c-myc antisense oligodeoxynucleotides on human melanoma (M14) cells Anticancer Res 17 1A 29 35 9066627

[31] Bielinska A 1996 Regulation of in vitro gene expression using antisense oligonucleotides or antisense expression plasmids transfected using starburst PAMAM dendrimers Nucleic Acids Res 24 11 2176 82 8668551 10.1093/nar/24.11.2176 PMC145901

[32] Delong R 1997 Characterization of complexes of oligonucleotides with polyamidoamine starburst dendrimers and effects on intracellular delivery J. Pharm. Sci 86 6 762 4 9188063 10.1021/js960409f

[33] Morris MC 1997 A new peptide vector for efficient delivery of oligonucleotides into mammalian cells Nucleic Acids Res 25 14 2730 6 9207018 10.1093/nar/25.14.2730 PMC146800

[34] Dokka S 1997 Cellular delivery of oligonucleotides by synthetic import peptide carrier Pharm. Res 14 12 1759 64 9453065 10.1023/a:1012188014919

[35] de La Torre BG 1999 Synthesis and binding properties of oligonucleotides carrying nuclear localization sequences Bioconjug. Chem 10 6 1005 12 10563769 10.1021/bc990046l

[36] Chavany C 1994 Adsorption of oligonucleotides onto polyisohexylcyanoacrylate nanoparticles protects them against nucleases and increases their cellular uptake Pharm. Res 11 9 1370 8 7816773 10.1023/a:1018923301967

[37] Spiller DG Tidd DM 1995 Nuclear delivery of antisense oligodeoxynucleotides through reversible permeabilization of human leukemia cells with streptolysin O Antisense Res. Dev 5 1 13 21 7613071 10.1089/ard.1995.5.13

[38] Zewert TE 1995 Transdermal transport of DNA antisense oligonucleotides by electroporation Biochem. Biophys. Res. Commun 212 2 286 92 7626040 10.1006/bbrc.1995.1968

[39] Huber PE 1999 A comparison of shock wave and sinusoidal-focused ultrasound-induced localized transfection of HeLa cells Ultrasound Med. Biol 25 9 1451 7 10626634 10.1016/s0301-5629(99)00099-x

[40] Stein CA Benimetskaya L Mani S 2005 Antisense strategies for oncogene inactivation Semin. Oncol 32 6 563 72 16338422 10.1053/j.seminoncol.2005.09.003

[41] Levin AA 1999 A review of the issues in the pharmacokinetics and toxicology of phosphorothioate antisense oligonucleotides Biochim. Biophys. Acta 1489 1 69 84 10806998 10.1016/s0167-4781(99)00140-2

[42] Geary RS Henry SP Grillone LR 2002 Fomivirsen: clinical pharmacology and potential drug interactions Clin. Pharmacokinet 41 4 255 60 11978144 10.2165/00003088-200241040-00002

[43] Iversen P 1991 In vivo studies with phosphorothioate oligonucleotides: pharmacokinetics prologue Anticancer Drug Des 6 6 531 8 1772568

[44] Grindel JM 1998 Pharmacokinetics and metabolism of an oligodeoxynucleotide phosphorothioate (GEM91) in cynomolgus monkeys following intravenous infusion Antisense Nucleic Acid Drug Dev 8 1 43 52 9512095 10.1089/oli.1.1998.8.43

[45] Henry SP 1997 Toxicological and pharmacokinetic properties of chemically modified antisense oligonucleotide inhibitors of PKC-alpha and C-raf kinase Anticancer Drug Des 12 5 409 20 9236856

[46] Butler M Stecker K Bennett CF 1997 Cellular distribution of phosphorothioate oligodeoxynucleotides in normal rodent tissues Lab Invest 77 4 379 88 9354772

[47] Lopes de Menezes DE Mayer LD 2002 Pharmacokinetics of Bcl-2 antisense oligonucleotide (G3139) combined with doxorubicin in SCID mice bearing human breast cancer solid tumor xenografts Cancer Chemother. Pharmacol 49 1 57 68 11855753 10.1007/s00280-001-0385-3

[48] Sewell KL 2002 Phase I trial of ISIS 104838, a 2′-methoxyethyl modified antisense oligonucleotide targeting tumor necrosis factor-alpha J. Pharmacol. Exp. Ther 303 3 1334 43 12438559 10.1124/jpet.102.036749

[49] Yu RZ 2004 Tissue disposition of 2′-O-(2-methoxy) ethyl modified antisense oligonucleotides in monkeys J. Pharm. Sci 93 1 48 59 14648635 10.1002/jps.10473

[50] Arora V 2002 Bioavailability and efficacy of antisense morpholino oligomers targeted to c-myc and cytochrome P-450 3A2 following oral administration in rats J. Pharm. Sci 91 4 1009 18 11948540 10.1002/jps.10088

[51] Arora V Devi GR Iversen PL 2004 Neutrally charged phosphorodiamidate morpholino antisense oligomers: uptake, efficacy and pharmacokinetics Curr. Pharm. Biotechnol 5 5 431 9 15544491 10.2174/1389201043376706

[52] Lebedeva I Stein CA 2001 Antisense oligonucleotides: promise and reality Annu. Rev. Pharmacol. Toxicol 41 403 19 11264463 10.1146/annurev.pharmtox.41.1.403

[53] Chan JH Lim S Wong WS 2006 Antisense oligonucleotides: from design to therapeutic application Clin. Exp. Pharmacol. Physiol 33 5–6 533 40 16700890 10.1111/j.1440-1681.2006.04403.x

[54] Burgess TL 1995 The antiproliferative activity of c-myb and c-myc antisense oligonucleotides in smooth muscle cells is caused by a nonantisense mechanism Proc. Natl. Acad. Sci. U.S.A 92 9 4051 5 7732029 10.1073/pnas.92.9.4051 PMC42100

[55] Lang R 1999 Guanosine-rich oligodeoxynucleotides induce proliferation of macrophage progenitors in cultures of murine bone marrow cells Eur. J. Immunol 29 11 3496 506 10556804 10.1002/(SICI)1521-4141(199911)29:11<3496::AID-IMMU3496>3.0.CO;2-3

[56] Krieg AM 1999 Mechanisms and applications of immune stimulatory CpG oligodeoxynucleotides Biochim. Biophys. Acta 1489 1 107 16 10807001 10.1016/s0167-4781(99)00147-5

[57] Krug A 2001 Toll-like receptor expression reveals CpG DNA as a unique microbial stimulus for plasmacytoid dendritic cells which synergizes with CD40 ligand to induce high amounts of IL-12 Eur. J. Immunol 31 10 3026 37 11592079 10.1002/1521-4141(2001010)31:10<3026::aid-immu3026>3.0.co;2-h

[58] Brazolot Millan CL Weeratna R Krieg AM Siegrist CA Davis HL 1998 CpG DNA can induce strong Th1 humoral and cell-mediated immune responses against hepatitis B. surface antigen in young mice Proc. Natl. Acad. Sci. U.S.A 95 26 15553 58 9861007 10.1073/pnas.95.26.15553 PMC28081

[59] Monteith DK 1998 Preclinical evaluation of the effects of a novel antisense compound targeting C-raf kinase in mice and monkeys Toxicol. Sci 46 2 365 75 10048140 10.1006/toxs.1998.2527

[60] Crooke ST 2004 Progress in antisense technology Annu. Rev. Med 55 61 95 14746510 10.1146/annurev.med.55.091902.104408

[61] Henry SP 1999 Correlation of toxicity and pharmacokinetic properties of a phosphorothioate oligonucleotide designed to inhibit ICAM-1 Toxicol. Pathol 27 1 95 100 10367680 10.1177/019262339902700117

[62] Zhou W Agrawal S 1998 Mixed-backbone oligonucleotides as second-generation antisense agents with reduced phosphorothioate-related side effects Bioorg Med. Chem. Lett 8 22 3269 74 9873716 10.1016/s0960-894x(98)00591-5

[63] Reynolds T 1992 First antisense drug trials planned in leukemia J. Natl. Cancer Inst 84 5 288 90 1738177 10.1093/jnci/84.5.288

[64] Wacheck V Zangemeister-Wittke U 2006 Antisense molecules for targeted cancer therapy Crit. Rev. Oncol. Hematol 59 1 65 73 16750913 10.1016/j.critrevonc.2005.10.004

[65] Whitesell L Rosolen A Neckers LM 1991 In vivo modulation of N-myc expression by continuous perfusion with an antisense oligonucleotide Antisense Res. Dev 1 4 343 50 1821655 10.1089/ard.1991.1.343

[66] Gleave ME Monia BP 2005 Antisense therapy for cancer Nat. Rev. Cancer 5 6 468 79 15905854 10.1038/nrc1631

[67] Pirollo KF 2003 Antisense therapeutics: from theory to clinical practice Pharmacol. Ther 99 1 55 77 12804699 10.1016/s0163-7258(03)00053-6

[68] Kitada S 1994 Reversal of chemoresistance of lymphoma cells by antisense-mediated reduction of bcl-2 gene expression Antisense Res. Dev 4 2 71 9 7950302 10.1089/ard.1994.4.71

[69] Cotter FE 1994 Antisense oligonucleotides suppress B-cell lymphoma growth in a SCID-hu mouse model Oncogene 9 10 3049 55 8084613

[70] G 3139 Augmerosen, Bcl-2 antisense oligonucleotide—Genta, GC 3139, Genasense Drugs R D3 1 44 9 10.2165/00126839-200203010-00010 11881530

[71] Waters JS 2000 Phase I clinical and pharmacokinetic study of bcl-2 antisense oligonucleotide therapy in patients with non-Hodgkin’s lymphoma J. Clin. Oncol 18 9 1812 23 10784621 10.1200/JCO.2000.18.9.1812

[72] Jansen B 2000 Chemosensitisation of malignant melanoma by BCL2 antisense therapy Lancet 356 9243 1728 33 11095261 10.1016/S0140-6736(00)03207-4

[73] Kirkwood JM Bedikian AY Millwars MJ 2005 Long-term survival results of a randomized multinational phase 3 of dacarbazine (DTIC) with or without bcl-2 antisense (oblimersen sodium)in patients with advanced malignant melanoma Proc. Am. Soc. Clin. Oncol 23 711

[74] Ways DK 1995 MCF-7 breast cancer cells transfected with protein kinase C-alpha exhibit altered expression of other protein kinase C isoforms and display a more aggressive neoplastic phenotype J. Clin. Invest 95 4 1906 15 7706498 10.1172/JCI117872 PMC295735

[75] Yazaki T 1996 Treatment of glioblastoma U-87 by systemic administration of an antisense protein kinase C-alpha phosphorothioate oligodeoxynucleotide Mol. Pharmacol 50 2 236 42 8700129

[76] McGraw K 1997 Antisense oligonucleotide inhibitors of isozymes of protein kinase C: in vitro and in vivo activity, and clinical development as anti-cancer therapeutics Anticancer Drug Des 12 5 315 26 9236849

[77] Geiger T 1998 Antitumor activity of a PKC-alpha antisense oligonucleotide in combination with standard chemotherapeutic agents against various human tumors transplanted into nude mice Anticancer Drug Des 13 1 35 45 9474241

[78] Nemunaitis J 1999 Phase I evaluation of ISIS 3521, an anti-sense oligodeoxynucleotide to protein kinase C-alpha, in patients with advanced cancer J. Clin. Oncol 17 11 3586 95 10550158 10.1200/JCO.1999.17.11.3586

[79] Yuen AR 1999 Phase I study of an antisense oligonucleotide to protein kinase C-alpha (ISIS 3521/CGP 64128A) in patients with cancer Clin. Cancer Res 5 11 3357 63 10589745

[80] Advani R 2005 A phase I trial of aprinocarsen (ISIS 3521/LY.900003), an antisense inhibitor of protein kinase C-alpha administered as a 24-hour weekly infusion schedule in patients with advanced cancer Invest. New Drugs 23 5 467 77 16133798 10.1007/s10637-005-2906-0

[81] Villalona-Calero MA 2004 A phase I/II study of LY.900003, an antisense inhibitor of protein kinase C-alpha, in combination with cisplatin and gemcitabine in patients with advanced non-small cell lung cancer Clin. Cancer Res 10 18 Pt 1 6086 93 15447994 10.1158/1078-0432.CCR-04-0779

[82] Marshall JL 2004 A phase II trial of ISIS 3521 in patients with metastatic colorectal cancer Clin. Colorectal Cancer 4 4 268 74 15555210 10.3816/ccc.2004.n.026

[83] Ghobrial IM Adjei AA 2002 Inhibitors of the ras oncogene as therapeutic targets Hematol. Oncol. Clin. North Am 16 5 1065 88 12512383 10.1016/s0889-8588(02)00050-3

[84] Chen G 1996 Antisense oligonucleotides demonstrate a dominant role of c-Ki-RAS proteins in regulating the proliferation of diploid human fibroblasts J. Biol. Chem 271 45 28259 65 8910444 10.1074/jbc.271.45.28259

[85] Cunningham CC 2001 A Phase I trial of H-ras antisense oligonucleotide ISIS 2503 administered as a continuous intravenous infusion in patients with advanced carcinoma Cancer 92 5 1265 71 11571742 10.1002/1097-0142(20010901)92:5<1265::aid-cncr1447>3.0.co;2-5

[86] Alberts SR 2004 Gemcitabine and ISIS-2503 for patients with locally advanced or metastatic pancreatic adenocarcinoma: a North Central Cancer Treatment Group phase II trial J. Clin. Oncol 22 24 4944 50 15611509 10.1200/JCO.2004.05.034

[87] Howe LR 1992 Activation of the MAP kinase pathway by the protein kinase raf Cell 71 2 335 42 1330321 10.1016/0092-8674(92)90361-f

[88] Wang HG Rapp UR Reed JC 1996 Bcl-2 targets the protein kinase Raf-1 to mitochondria Cell 87 4 629 38 8929532 10.1016/s0092-8674(00)81383-5

[89] Storm SM Rapp UR 1993 Oncogene activation: c-raf-1 gene mutations in experimental and naturally occurring tumors Toxicol. Lett 67 1–3 201 10 8451761 10.1016/0378-4274(93)90056-4

[90] Monia BP 1996 Antitumor activity of a phosphorothioate antisense oligodeoxynucleotide targeted against C-raf kinase Nat. Med 2 6 668 75 8640558 10.1038/nm0696-668

[91] Gokhale PC 1999 Antisense raf oligodeoxyribonucleotide is a radiosensitizer in vivo Antisense Nucleic Acid Drug Dev 9 2 191 201 10355825 10.1089/oli.1.1999.9.191

[92] O’Dwyer PJ 1999 c-raf-1 depletion and tumor responses in patients treated with the c-raf-1 antisense oligodeoxynucleotide ISIS 5132 (CGP 69846A) Clin. Cancer Res 5 12 3977 82 10632328

[93] Cunningham CC 2000 A phase I trial of c-Raf kinase antisense oligonucleotide ISIS 5132 administered as a continuous intravenous infusion in patients with advanced cancer Clin. Cancer Res 6 5 1626 31 10815879

[94] Stevenson JP 1999 Phase I clinical/pharmacokinetic and pharmacodynamic trial of the c-raf-1 antisense oligonucleotide ISIS 5132 (CGP 69846A) J. Clin. Oncol 17 7 2227 36 10561280 10.1200/JCO.1999.17.7.2227

[95] Rudin CM 2001 Phase I Trial of ISIS 5132, an antisense oligonucleotide inhibitor of c-raf-1, administered by 24-hour weekly infusion to patients with advanced cancer Clin. Cancer Res 7 5 1214 20 11350886

[96] Tolcher AW 2002 A randomized phase II and pharmacokinetic study of the antisense oligonucleotides ISIS 3521 and ISIS 5132 in patients with hormone-refractory prostate cancer Clin. Cancer Res 8 8 2530 5 12171880

[97] Cripps MC 2002 Phase II randomized study of ISIS 3521 and ISIS 5132 in patients with locally advanced or metastatic colorectal cancer: a National Cancer Institute of Canada clinical trials group study Clin. Cancer Res 8 7 2188 92 12114419

[98] Oza AM 2003 Phase II study of CGP 69846A (ISIS 5132) in recurrent epithelial ovarian cancer: an NCIC clinical trials group study (NCIC IND.116) Gynecol. Oncol 89 1 129 33 12694666 10.1016/s0090-8258(02)00144-0

[99] Coudert B 2001 Phase II trial with ISIS 5132 in patients with small-cell (SCLC) and non-small cell (NSCLC) lung cancer. A European Organization for Research and Treatment of Cancer (EORTC) Early Clinical Studies Group report Eur. J. Cancer 37 17 2194 8 11677106 10.1016/s0959-8049(01)00286-6

[100] Gokhale PC 1997 Antisense raf oligodeoxyribonucleotide is protected by liposomal encapsulation and inhibits Raf-1 protein expression in vitro and in vivo: implication for gene therapy of radioresistant cancer Gene Ther 4 12 1289 99 9472552 10.1038/sj.gt.3300543

[101] Gokhale PC 2002 Pharmacokinetics, toxicity, and efficacy of ends-modified raf antisense oligodeoxyribonucleotide encapsulated in a novel cationic liposome Clin. Cancer Res 8 11 3611 21 12429653

[102] Pei J 2004 Combination with liposome-entrapped, ends-modified raf antisense oligonucleotide (LErafAON.) improves the anti-tumor efficacies of cisplatin, epirubicin, mitoxantrone, docetaxel and gemcitabine Anticancer Drugs 15 3 243 53 15014358 10.1097/00001813-200403000-00009

[103] Kasid U Dritschilo A 2003 RAF antisense oligonucleotide as a tumor radiosensitizer Oncogene 22 37 5876 84 12947394 10.1038/sj.onc.1206700

[104] Rudin CM 2004 Delivery of a liposomal c-raf-1 antisense oligonucleotide by weekly bolus dosing in patients with advanced solid tumors: a phase I study Clin. Cancer Res 10 21 7244 51 15534098 10.1158/1078-0432.CCR-04-0642

[105] Dritschilo A 2006 Phase I study of liposome-encapsulated c-raf antisense oligodeoxyribonucleotide infusion in combination with radiation therapy in patients with advanced malignancies Clin. Cancer Res 12 4 1251 9 16489081 10.1158/1078-0432.CCR-05-1260

[106] Nichols WG Boeckh M 2000 Recent advances in the therapy and prevention of CMV infections J. Clin. Virol 16 1 25 40 10680738 10.1016/s1386-6532(99)00065-7

[107] Jabs DA Griffiths PD 2002 Fomivirsen for the treatment of cytomegalovirus retinitis Am. J. Ophthalmol 133 4 552 6 11931791 10.1016/s0002-9394(02)01325-9

[108] Azad RF 1993 Antiviral activity of a phosphorothioate oligonucleotide complementary to RNA of the human cytomegalovirus major immediate-early region Antimicrob. Agents Chemother 37 9 1945 54 8239610 10.1128/aac.37.9.1945 PMC188097

[109] Anderson KP 1996 Inhibition of human cytomegalovirus immediate-early gene expression by an antisense oligonucleotide complementary to immediate-early RNA Antimicrob. Agents Chemother 40 9 2004 11 8878571 10.1128/aac.40.9.2004 PMC163463

[110] Highleyman L 1998 Fomivirsen Beta 31 29 11365261

[111] Grillone LR Lanz R 2001 Fomivirsen Drugs Today (Barc) 37 4 245 55 12768225 10.1358/dot.2001.37.4.620590

[112] Fomivirsen approved for CMV retinitis: first antisense drug AIDS Treat News 1998 302 7 11365764

[113] Kim SG 1995 Antiviral effect of phosphorothioate oligodeoxyribonucleotides complementary to human immunodeficiency virus Bioorg. Med. Chem 3 1 49 54 8612046 10.1016/0968-0896(94)00142-P PMC9212667

[114] Suzuki J 2002 Inhibition of human immunodeficiency virus type 1 activity in vitro by a new self-stabilized oligonucleotide with guanosine-thymidine quadruplex motifs J. Virol 76 6 3015 22 11861867 10.1128/JVI.76.6.3015-3022.2002 PMC135965

[115] Matsukura M 1989 Regulation of viral expression of human immunodeficiency virus in vitro by an antisense phosphorothioate oligodeoxynucleotide against rev (art/trs) in chronically infected cells Proc. Natl. Acad. Sci. U.S.A 86 11 4244 8 2471199 10.1073/pnas.86.11.4244 PMC287427

[116] Yamaguchi K 1997 The multiple inhibitory mechanisms of GEM 91, a gag antisense phosphorothioate oligonucleotide, for human immunodeficiency virus type 1 AIDS Res. Hum. Retroviruses 13 7 545 54 9135872 10.1089/aid.1997.13.545

[117] Sereni D 1999 Pharmacokinetics and tolerability of intravenous trecovirsen (GEM 91), an antisense phosphorothioate oligonucleotide, in HIV-positive subjects J. Clin. Pharmacol 39 1 47 54 9987700 10.1177/00912709922007552

[118] Zheng R 1999 Technology evaluation: GEM-92, Hybridon Inc Curr. Opin. Mol. Ther 1 4 521 3 11713769

[119] Tan SL 2002 Hepatitis C therapeutics: current status and emerging strategies Nat. Rev. Drug Discov 1 11 867 81 12415247 10.1038/nrd937

[120] Brown-Driver V 1999 Inhibition of translation of hepatitis C virus RNA by 2-modified antisense oligonucleotides Antisense Nucleic Acid Drug Dev 9 2 145 54 10355821 10.1089/oli.1.1999.9.145

[121] Alt M 1995 Specific inhibition of hepatitis C viral gene expression by antisense phosphorothioate oligodeoxynucleotides Hepatology 22 3 707 17 7657273

[122] Zhang H 1999 Antisense oligonucleotide inhibition of hepatitis C virus (HCV) gene expression in livers of mice infected with an HCV-vaccinia virus recombinant Antimicrob. Agents Chemother 43 2 347 53 9925530 10.1128/aac.43.2.347 PMC89075

[123] Witherell GW 2001 ISIS-14803 (Isis Pharmaceuticals) Curr. Opin. Investig. Drugs 2 11 1523 9 11763152

[124] McHutchison JG 2006 A phase I trial of an antisense inhibitor of hepatitis C virus (ISIS 14803), administered to chronic hepatitis C patients J. Hepatol 44 1 88 96 16274834 10.1016/j.jhep.2005.09.009

[125] Simmons D Makgoba MW Seed B 1988 ICAM, an adhesion ligand of LFA-1, is homologous to the neural cell adhesion molecule NCAM Nature 331 6157 624 7 3340213 10.1038/331624a0

[126] Butcher EC 1991 Leukocyte-endothelial cell recognition: three (or more) steps to specificity and diversity Cell 67 6 1033 6 1760836 10.1016/0092-8674(91)90279-8

[127] Bennett CF 1994 Inhibition of endothelial cell adhesion molecule expression with antisense oligonucleotides J. Immunol 152 7 3530 40 7511650

[128] Gewirtz AT Sitaraman SV 2005 Technology evaluation: alicaforsen (Isis) Curr. Opin. Mol. Ther 7 3 273 81 15977426

[129] Yacyshyn BR 1998 A placebo-controlled trial of ICAM-1 antisense oligonucleotide in the treatment of Crohn’s disease Gastroenterology 114 6 1133 42 9609749 10.1016/s0016-5085(98)70418-4

[130] Schreiber S 2001 Absence of efficacy of subcutaneous anti-sense ICAM-1 treatment of chronic active Crohn’s disease Gastroenterology 120 6 1339 46 11313303 10.1053/gast.2001.24015

[131] Yacyshyn BR 2002 Double blind, placebo controlled trial of the remission inducing and steroid sparing properties of an ICAM-1 antisense oligodeoxynucleotide, alicaforsen (ISIS 2302), in active steroid dependent Crohn’s disease Gut 51 1 30 6 12077088 10.1136/gut.51.1.30 PMC1773277

[132] van Deventer SJ Tami JA Wedel MK 2004 A randomised, controlled, double blind, escalating dose study of alicaforsen enema in active ulcerative colitis Gut 53 11 1646 51 15479686 10.1136/gut.2003.036160 PMC1774281

[133] Miner P 2004 An enema formulation of alicaforsen, an anti-sense inhibitor of intercellular adhesion molecule-1, in the treatment of chronic, unremitting pouchitis Aliment Pharmacol. Ther 19 3 281 6 14984374 10.1111/j.1365-2036.2004.01863.x

[134] Miner PB 2006 Safety and efficacy of two dose formulations of alicaforsen enema compared with mesalazine enema for treatment of mild to moderate left-sided ulcerative colitis: a randomized, double-blind, active-controlled trial Aliment Pharmacol. Ther 23 10 1403 13 16669955 10.1111/j.1365-2036.2006.02837.x

[135] van Deventer SJ 2006 A phase II dose ranging, double-blind, placebo-controlled study of alicaforsen enema in subjects with acute exacerbation of mild to moderate left-sided ulcerative colitis Aliment Pharmacol. Ther 23 10 1415 25 16669956 10.1111/j.1365-2036.2006.02910.x

[136] Miner PB 2006 Bioavailability and therapeutic activity of alicaforsen (ISIS 2302) administered as a rectal retention enema to subjects with active ulcerative colitis Aliment Pharmacol. Ther 23 10 1427 34 16669957 10.1111/j.1365-2036.2006.02909.x

[137] Kahan BD 2004 Phase I and phase II safety and efficacy trial of intercellular adhesion molecule-1 antisense oligodeoxynucleotide (ISIS 2302) for the prevention of acute allograft rejection Transplantation 78 6 858 63 15385805 10.1097/01.tp.0000128857.77893.d2

[138] Kast RE 2005 Evidence of a mechanism by which etanercept increased TNF-alpha in multiple myeloma: new insights into the biology of TNF-alpha giving new treatment opportunities—the role of bupropion Leuk Res 29 12 1459 63 15964626 10.1016/j.leukres.2005.05.006

[139] Kennewell P 2003 Technology evaluation: ISIS-104838, OraSense Curr. Opin. Mol. Ther 5 1 76 80 12669475

[140] Holmlund JT 2003 Applying antisense technology: Affinitak and other antisense oligonucleotides in clinical development Ann. N. Y. Acad. Sci 1002 244 51 14751839 10.1196/annals.1281.027

[141] Spicuzza L Di Maria G Polosa R 2006 Adenosine in the airways: implications and applications Eur. J. Pharmacol 533 1–3 77 88 16458886 10.1016/j.ejphar.2005.12.056

[142] Bjorck T Gustafsson LE Dahlen SE 1992 Isolated bronchi from asthmatics are hyperresponsive to adenosine, which apparently acts indirectly by liberation of leukotrienes and histamine Am. Rev. Respir. Dis 145 5 1087 91 1375009 10.1164/ajrccm/145.5.1087

[143] Ali S Mustafa SJ Metzger WJ 1994 Adenosine-induced bronchoconstriction and contraction of airway smooth muscle from allergic rabbits with late-phase airway obstruction: evidence for an inducible adenosine A1 receptor J. Pharmacol. Exp. Ther 268 3 1328 34 8138947

[144] Nyce JW 1999 Insight into adenosine receptor function using antisense and gene-knockout approaches Trends Pharmacol. Sci 20 2 79 83 10101969 10.1016/s0165-6147(99)01305-x

[145] Tanaka M Nyce JW 2001 Respirable antisense oligonucleotides: a new drug class for respiratory disease Respir. Res 2 1 5 9 11686859 10.1186/rr32 PMC59563

[146] Zhang Z 2002 Technology evaluation: EPI-2010, EpiGenesis Curr. Opin. Mol. Ther 4 3 275 80 12139314

[147] Nyce JW Metzger WJ 1997 DNA antisense therapy for asthma in an animal model Nature 385 6618 721 5 9034188 10.1038/385721a0

[148] Ali S 2001 Absorption, distribution, metabolism, and excretion of a respirable antisense oligonucleotide for asthma Am. J. Respir. Crit. Care Med 163 4 989 93 11282778 10.1164/ajrccm.163.4.9907078

[149] Ball HA Van Scott MR Robinson CB 2004 Sense and antisense: therapeutic potential of oligonucleotides and interference RNA in asthma and allergic disorders Clin. Rev. Allergy Immunol 27 3 207 17 15630157 10.1385/CRIAI:27:3:207

[150] Egner U 2005 The target discovery process Chembiochem 6 3 468 79 15742383 10.1002/cbic.200400158

[151] Borsch-Haubold AG Pasquet S Watson SP 1998 Direct inhibition of cyclooxygenase-1 and -2 by the kinase inhibitors SB. 203580 and PD 98059. SB. 203580 also inhibits thromboxane synthase J. Biol. Chem 273 44 28766 72 9786874 10.1074/jbc.273.44.28766

[152] Taylor MF 2001 Emerging antisense technologies for gene functionalization and drug discovery DDT 6 15 (Suppl) S97 101

[153] Zhi L Leung BP Melendez AJ 2006 Sphingosine kinase 1 regulates pro-inflammatory responses triggered by TNFalpha in primary human monocytes J. Cell. Physiol 208 1 109 15 16575915 10.1002/jcp.20646

[154] Melendez AJ Ibrahim FB 2004 Antisense knockdown of sphingosine kinase 1 in human macrophages inhibits C5a receptor-dependent signal transduction, Ca2+ signals, enzyme release, cytokine production, and chemotaxis J. Immunol 173 3 1596 603 15265887 10.4049/jimmunol.173.3.1596

[155] Alifano P 1994 Ribonuclease E provides substrates for ribonuclease P-dependent processing of a polycistronic mRNA Genes Dev 8 24 3021 31 8001821 10.1101/gad.8.24.3021

[156] Rossi JJ Gewirtz AM Therapeutic Applications of ribozymes Nucleic acid therapeutics in cancer 2004 New Jersey Humana Press 45 8

[157] Haseloff J Gerlach WL 1988 Simple RNA enzymes with new and highly specific endoribonuclease activities Nature 334 6183 585 91 2457170 10.1038/334585a0

[158] Sarver N 1990 Ribozymes as potential anti-HIV-1 therapeutic agents Science 247 4947 1222 5 2107573 10.1126/science.2107573

[159] Pavco PA 2000 Antitumor and antimetastatic activity of ribozymes targeting the messenger RNA of vascular endothelial growth factor receptors Clin. Cancer Res 6 5 2094 103 10815937

[160] Ngok FK 2004 Clinical gene therapy research utilizing ribozymes: application to the treatment of HIV/AIDS Methods Mol. Biol 252 581 98 15017082 10.1385/1-59259-746-7:581

[161] Weng DE 2005 A phase I clinical trial of a ribozyme-based angiogenesis inhibitor targeting vascular endothelial growth factor receptor-1 for patients with refractory solid tumors Mol. Cancer Ther 4 6 948 55 15956252 10.1158/1535-7163.MCT-04-0210

[162] Elbashir SM 2001 Duplexes of 21-nucleotide RNAs mediate RNA interference in cultured mammalian cells Nature 411 6836 494 8 11373684 10.1038/35078107

[163] Lu PY Xie FY Woodle MC 2003 siRNA-mediated antitumorigenesis for drug target validation and therapeutics Curr. Opin. Mol. Ther 5 3 225 34 12870431

[164] Reich SJ 2003 Small interfering RNA (siRNA) targeting VEGF effectively inhibits ocular neovascularization in a mouse model Mol. Vis 9 210 6 12789138

[165] Frantz S 2006 Safety concerns raised over RNA interference Nat. Rev. Drug Discov 5 7 528 9 16883643 10.1038/nrd2104

[166] Bartel DP 2004 MicroRNAs: genomics, biogenesis, mechanism, and function Cell 116 2 281 97 14744438 10.1016/s0092-8674(04)00045-5

[167] Dugas DV Bartel B 2004 MicroRNA regulation of gene expression in plants Curr. Opin. Plant Biol 7 5 512 20 15337093 10.1016/j.pbi.2004.07.011

[168] Lee RC Feinbaum RL Ambros V 1993 The C. elegans heterochronic gene lin-4 encodes small RNAs with antisense complementarity to lin-14 Cell 75 5 843 54 8252621 10.1016/0092-8674(93)90529-y

[169] Reinhart BJ 2000 The 21 nucleotide let-7 RNA regulates developmental timing in Caenorhabditis elegans Nature 403 6772 901 06 10706289 10.1038/35002607

[170] Carrington JC Ambros V 2003 Role of microRNAs in plant and animal development Science 301 5631 336 8 12869753 10.1126/science.1085242

[171] Phillips MI Phillips MI Antisense therapeutics. A promise waiting to be fulfilled 2nd ed Antisense Therapeutics 2005 New Jersey Humana Press 15375309

[172] Frantz S 2004 Lessons learnt from Genasense’s failure Nat. Rev. Drug Discov 3 7 542 3 15272494 10.1038/nrd1464

